# Methyl Donor Deficiency during Gestation and Lactation in the Rat Affects the Expression of Neuropeptides and Related Receptors in the Hypothalamus

**DOI:** 10.3390/ijms20205097

**Published:** 2019-10-14

**Authors:** Lynda Saber Cherif, Grégory Pourié, Andréa Geoffroy, Amélia Julien, Déborah Helle, Aurélie Robert, Rémy Umoret, Jean-Louis Guéant, Carine Bossenmeyer-Pourié, Jean-Luc Daval

**Affiliations:** Inserm U1256, Faculté de Médecine, Université de Lorraine, 9 avenue de la Forêt de Haye, F-54500 Vandoeuvre-lès-Nancy, France; lynda.saber-cherif@univ-lorraine.fr (L.S.C.); gregory.pourie@univ-lorraine.fr (G.P.); g.andrea@hotmail.fr (A.G.); amelia.julien@univ-lorraine.fr (A.J.); deborah.helle@univ-lorraine.fr (D.H.); aurelie.robert@univ-lorraine.fr (A.R.); remy.umoret@univ-lorraine.fr (R.U.); jean-louis.gueant@univ-lorraine.fr (J.-L.G.)

**Keywords:** development, growth, hypothalamus, energy homeostasis, orexigenic and anorexigenic pathways, gestational deficiency, folate, vitamin B12, folate supplementation

## Abstract

The micronutrients vitamins B9 and B12 act as methyl donors in the one-carbon metabolism involved in transmethylation reactions which critically influence epigenetic mechanisms and gene expression. Both vitamins are essential for proper development, and their deficiency during pregnancy has been associated with a wide range of disorders, including persisting growth retardation. Energy homeostasis and feeding are centrally regulated by the hypothalamus which integrates peripheral signals and acts through several orexigenic and anorexigenic mediators. We studied this regulating system in a rat model of methyl donor deficiency during gestation and lactation. At weaning, a predominance of the anorexigenic pathway was observed in deficient pups, with increased plasma peptide YY and increased hypothalamic pro-opiomelanocortin (POMC) mRNA, in line with abnormal leptin, ghrelin, and insulin secretion and/or signaling during critical periods of fetal and/or postnatal development of the hypothalamus. These results suggest that early methyl donor deficiency can affect the development and function of energy balance circuits, resulting in growth and weight deficits. Maternal administration of folic acid (3 mg/kg/day) during the perinatal period tended to rectify peripheral metabolic signaling and central neuropeptide and receptor expression, leading to reduced growth retardation.

## 1. Introduction

The nutritional methyl donors folate (vitamin B9) and vitamin B12 are cofactors in the one-carbon metabolism that plays a critical role in transmethylation reactions [1,2], and are key regulators of the concentration of homocysteine which, if present in excess, can exert adverse effects such as DNA breakage, oxidative stress, endoplasmic reticulum stress, protein homocysteinylation, and apoptosis [3,4,5,6]. Both vitamins are essential for normal development, and their insufficiency constitutes a risk factor for various developmental disorders, particularly neural tube defects [7,8,9]. By using a validated rat model of methyl donor deficiency during the gestational and lactating periods, previous studies in our laboratory have shown that folate and vitamin B12 deprivation was associated in the offspring with persisting global growth retardation, brain atrophy, and morphological and functional defects [10,11,12]. Whereas the stomach size in deficient pups was found to be much smaller than in control animals at the same age, concomitantly with severe gastritis, a dysfunction of the ghrelin system was also demonstrated [13]. This gastrointestinal hormone plays a significant role in growth through its dual role as a growth hormone-releasing factor and as an appetite-stimulating peptide through hypothalamic actions [14]. The hypothalamus is composed of several nuclei that produce neuropeptides involved in key physiological functions. Feeding behavior is finely tuned by the interactions of circulating hormones with peripheral peptidergic and aminergic projections that arise from the brainstem and midbrain to the hypothalamus, and ghrelin could inhibit hypothalamic serotonin release [15,16]. The arcuate nucleus plays a role in food intake and energy homoeostasis regulation. It integrates peripheral signals such as circulating hormones (e.g., ghrelin, leptin, insulin, peptide YY) and nutrients, and contains two types of neurons. One type co-expresses neuropeptide Y (NPY) and agouti-related protein (AgRP) that stimulate food intake, while the other set of neurons co-expresses pro-opiomelanocortin (POMC) and cocaine- and amphetamine-regulated transcript (CART), which reduce food intake. Both populations project to the paraventricular nucleus and other areas important in the regulation of appetite [17,18,19,20]. Leptin and insulin act on their respective receptors, Ob-Rb and insulin receptor, to reduce the expression and release of hypothalamic orexigenic peptides and stimulate anorexigenic peptides. Recently, we showed in our experimental model that methyl donor deficiency disrupts brain Stat3 signaling pathway [11], which is required for hypothalamic regulation of energy balance by leptin through its receptor [21].

Given our previous observations and according to the concept stating that an adverse perinatal environment programs the development of several tissues, with long-term consequences on physiology and health [22,23], we hypothesized that exposure to methyl donor deficiency during the fetal period and in early life may alter the normal development and function of the hypothalamus, resulting in energy balance dysfunction and reduced growth. We therefore studied the hypothalamic expression profiles of neuropeptides and some related receptors in our rat model of methyl donor deficiency as well as in two hypothalamic cell lines deprived in B9: one mouse cell line (mHypoE46) expressing NPY, AgRP, leptin receptor, insulin receptor and insulin growth factor receptor 1 (Igf1R), and one rat cell line (rHypo-E11) expressing ghrelin, neuropeptide Y receptor 1 (NPY1R), leptin receptor, insulin receptor and Igf1R. The mouse cell line therefore displays the characteristics of NPY/AgRP neuronal cells of the arcuate nucleus, whereas the rat cell line mimics the ghrelin-containing regulating cells [24].

In addition, we investigated whether a perinatal supplementation with folic acid is able to reduce growth retardation in deprived rats and its influence on neuropeptide expression.

Taken together, our data showed that in utero and early postnatal deficiency in folate and vitamin B12 was associated with growth retardation, in line with misexpression of hypothalamic neuropeptides and related receptors regulating energy homeostasis, suggesting impaired development of hypothalamic networks. Importantly, perinatal and postnatal supplementation with folic acid helped restore mRNA levels and could be an effective strategy to reduce deficiency-related defects.

## 2. Results

### 2.1. Effects of Methyl Donor Deficiency on the Expression of Neuropeptides and Receptors in Mouse and Rat Embryonic Hypothalamic Cell Lines

**Figure 1:**
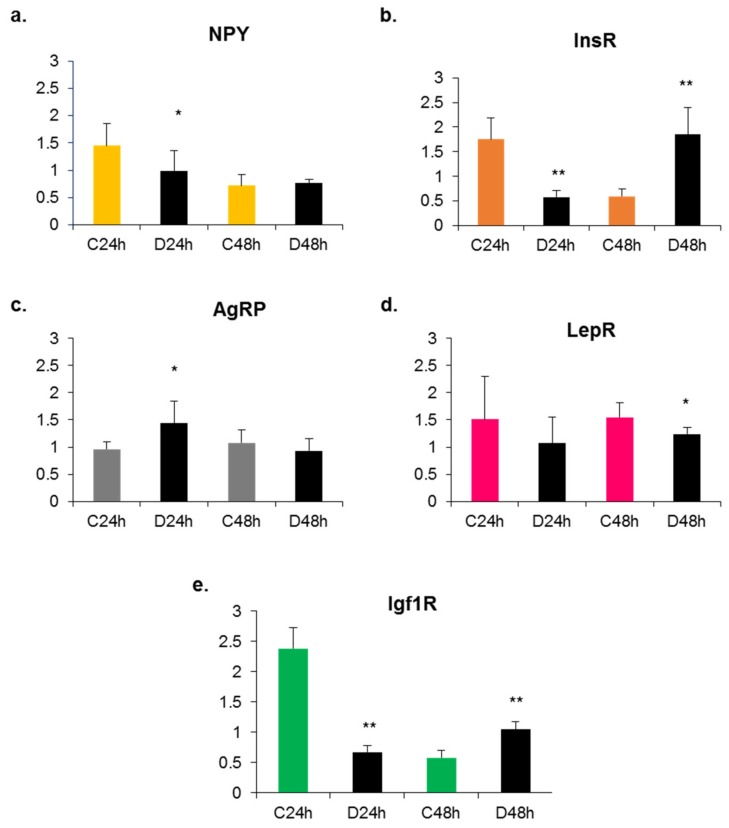
Effects of methyl donor deficiency on mRNA expression of neuropeptides and receptors in the mouse cell line (mHypoE46) at 24 and 48 h of growth. Data are means ± SD from 6 ≤ *n* ≤ 8 samples and are reported in arbitrary units. Statistically significant differences between control (C) and B9 deficient (D) cells: * *p* < 0.05 and ** *p* < 0.01. (**a**) Neuropeptide Y (NPY); (**b**) Insulin receptor (InsR); (**c**) Agouti-related protein (AgRP); (**d**) Leptin receptor (LepR); (**e**) Insulin growth factor receptor 1 (Igf1R).

**Figure 2:**
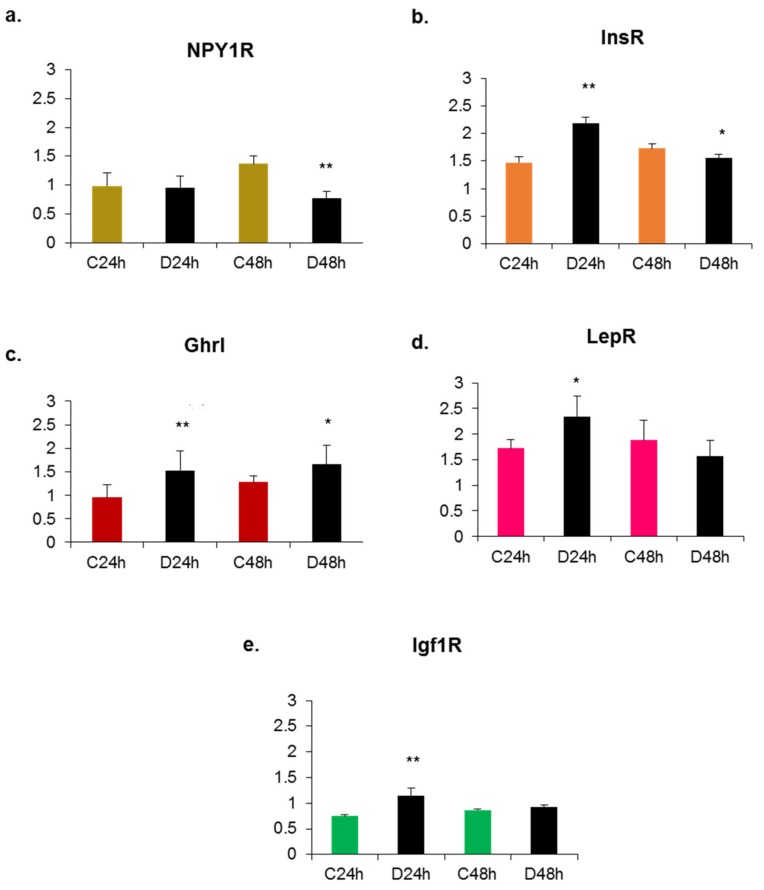
Effects of methyl donor deficiency on mRNA expression of neuropeptides and receptors in the rat cell line (rHypoE11) at 24 and 48 h of growth. Data are means ± SD from 6 ≤ *n* ≤ 8 samples and are reported in arbitrary units. Statistically significant differences between control (C) and B9 deficient (D) cells: * *p* < 0.05 and ** *p* < 0.01. (**a**) Neuropeptide Y receptor 1 (NPY1R); (**b**) insulin receptor (InsR); (**c**) ghrelin (Ghrl); (**d**) leptin receptor (LepR); (**e**) insulin growth factor receptor 1 (Igf1R).

The profiles of expression changes in response to B9 deprivation reflect the specificities of mouse and rat cell lines (Figure 1 and Figure 2).

RT-qPCR experiments led to two striking observations: the expression patterns of neuropeptides and receptors differed between the two cell lines on the one hand, and on the other, they varied as a function of time in a given cell line. We observed that the rat cell line is a hypothalamic cell line secreting ghrelin and expressing receptors for NPY, insulin and leptin whereas the mouse cell line is an NPY/AgRP-secreting cell line. After 24 h of B9 deficiency, the expression of the AgRP gene was increased in the mouse cell line while the expression of NPY and of the various receptors was decreased. In the rat cell line, a 24 h B9 deficiency induced an overexpression of the genes encoding ghrelin and the different receptors. These results reflect the direct effect of B9 deficiency on hypothalamic neuropeptides and receptors’ gene expression and show that receptor expression varied depending on the neuropeptide secretion by the cells.

### 2.2. Plasma Concentrations of Folate, Vitamin B12 and Homocysteine, and Growth Status of Weaned Rat Pups

As previously documented, nutritional methyl donor deficiency starting 1 month prior to mating affected female ability to conceive. Globally, 45.7% gave birth to pups (vs. 84% in controls). The number of live fetuses per litter was consistently reduced (6.7 vs. 11.2) [25]. In addition to spontaneous abortions, maternal pup-killing behavior and cannibalism were more frequently observed in deficient dams, as previously documented for thiamine deficiency [26]. Maternal supplementation with folic acid by itself had no significant effects on the same parameters.

In response to maternal deficiency during gestation and lactation, plasma levels of folate and vitamin B12 were dramatically reduced in the rat progeny at weaning (postnatal day 21). In parallel, homocysteinemia was significantly augmented (*p* < 0.01). Table 1 shows that folic acid supplementation restored folate concentration without affecting vitamin B12 status, and significantly reduced hyperhomocysteinemia in deficient pups.

In rat pups born to deficient dams, body weight was decreased by 55% as compared to controls at 21 days of age, whereas brain weight was concomitantly reduced by 30%. Growth was noticeably ameliorated by maternal supplementation with folic acid (Table 1).

### 2.3. Plasma Concentrations of Peripheral Hormones

**Figure 3:**
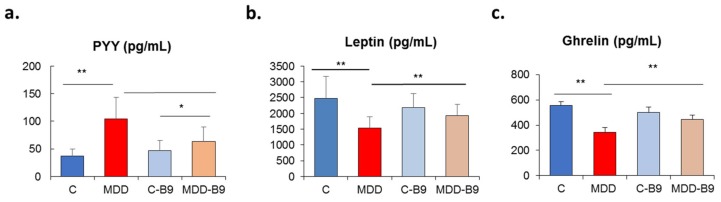
Effects of methyl donor deficiency on plasma concentrations of peripheral hormones rat pups at 21 days of age, and effects of folate supplementation. (**a**) Peptide YY (PYY); (**b**) leptin; (**c**) ghrelin. Data are means ± SD from six individuals per group. Statistically significant differences between the different animal groups: * *p* < 0.05 and ** *p* < 0.01.

Figure 3 shows plasma levels of peripheral hormones playing a role in appetite control, i.e., ghrelin, leptin and peptide YY (peptide tyrosine tyrosine). The concentration of the anorexigenic peptide YY was dramatically increased in the plasma of deficient pups (Figure 3a), suggesting satiety and decreased food intake. By contrast, plasma levels of leptin and ghrelin were decreased, reflecting a dysregulation of the hormone tandem. In all cases, maternal folate supplementation tended to remediate the situation.

### 2.4. Effects of Methyl Donor Deficiency on the Expression of Neuropeptides and Receptors in the Hypothalamus of Rat Pups—Consequences of Maternal Folate Supplementation

**Figure 4:**
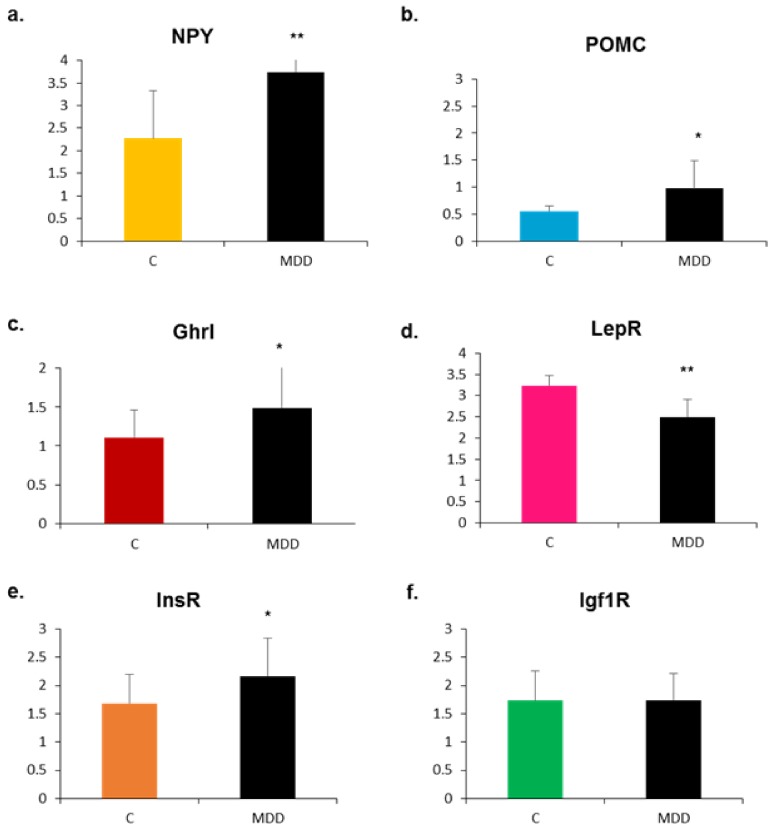
Effects of methyl donor deficiency on mRNA expression of neuropeptides and receptors in the hypothalamus of rat pups at 21 days of age. Data are means ± SD from 6 ≤ *n* ≤ 8 individuals and are reported in arbitrary units. Statistically significant differences between control (C) and deficient (MDD) rats: * *p* < 0.05 and ** *p* < 0.01. (**a**) Neuropeptide Y (NPY); (**b**) pro-opiomelanocortin (POMC), (**c**) ghrelin (Ghrl); (**d**) leptin receptor (LepR); (**e**) insulin receptor (InsR); (**f**) insulin growth factor receptor 1 (Igf1R).

As shown in Figure 4, the expression of all genes studied was enhanced following exposure to methyl donor deficiency, except for insulin growth factor receptor 1 (Igf1R), which remained unaffected (Figure 4f).

**Figure 5:**
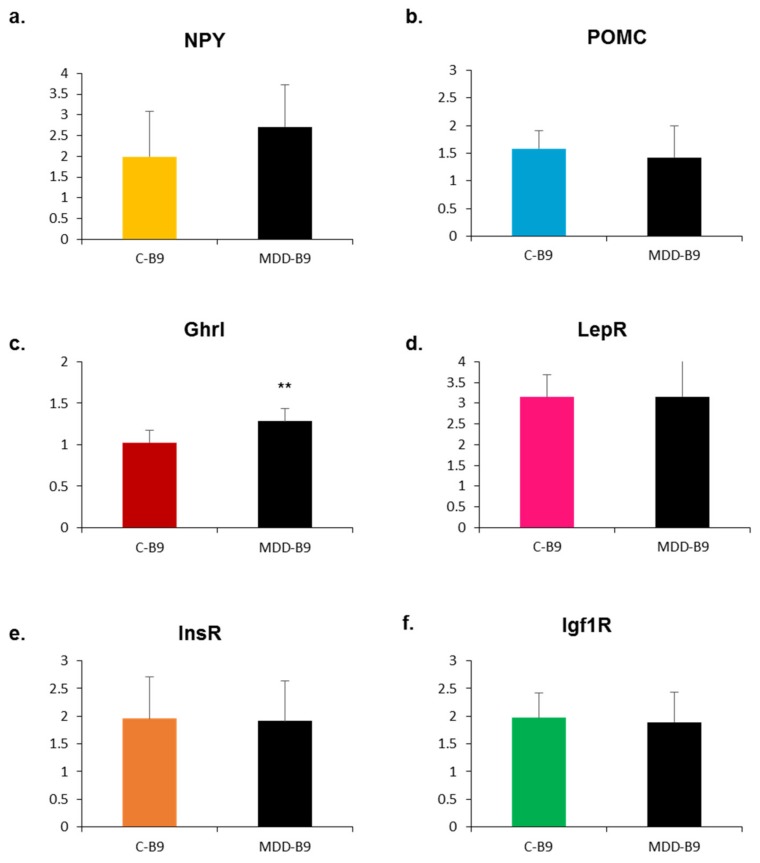
Effects of folate supplementation on mRNA expression of neuropeptides and receptors in the hypothalamus of rat pups at 21 days of age. Data are means ± SD from 6 ≤ *n* ≤ 8 individuals and are reported in arbitrary units. Statistically significant differences between control (C-B9) and deficient (MDD-B9) rats: ** *p* < 0.01. (**a**) Neuropeptide Y (NPY); (**b**) pro-opiomelanocortin (POMC); (**c**) ghrelin (Ghrl); (**d**) leptin receptor (LepR); (**e**) insulin receptor (InsR); (**f**) insulin growth factor receptor 1 (Igf1R).

Interestingly, maternal supplementation with folic acid restored mRNA expression levels which were affected by methyl donor deficiency in the hypothalamus of rat pups (Figure 5), except for the ghrelin gene that remained significantly more elevated than control (Figure 5c).

**Figure 6:**
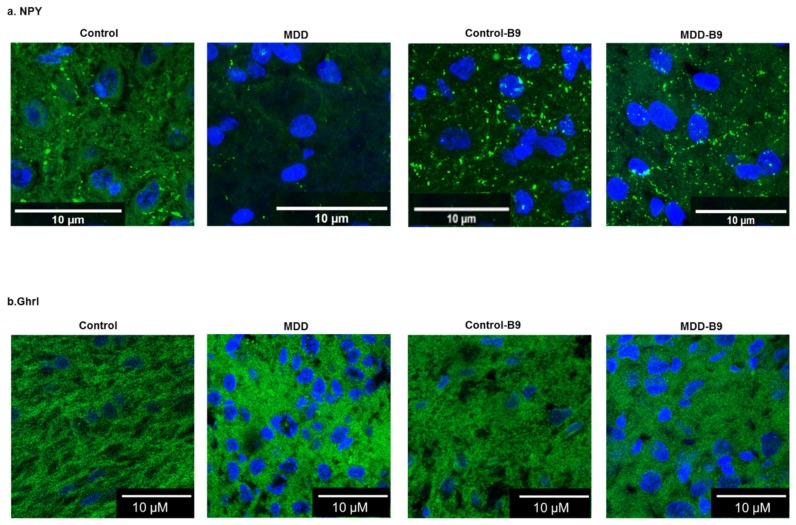
Effects of methyl donor deficiency and folate supplementation on the immunohistochemical expression of neuropeptide Y and ghrelin in the arcuate nucleus of the hypothalamus of control and deficient (MDD) rat pups at 21 days. (**a**) Neuropeptide Y (NPY); (**b**) ghrelin (Ghrl). Cell nuclei are counterstained by DAPI.

Figure 6 illustrates the expression of NPY and ghrelin in the arcuate nucleus in the various experimental conditions. The punctuate labeling of NPY expression reflects its high localization in axonal projections. The observation contrasts with methyl donor deficiency-associated NPY mRNA levels, suggesting a post-transcriptional regulation.

## 3. Discussion

In our animal model, maternal exposure to methyl donor deficiency is associated with a global growth retardation and brain atrophy in the offspring. In a previous study using the same experimental conditions, we mentioned that femur length, reflecting pre- and postnatal growth, was reduced by 21% at 21 days of age [27]. While normalizing the one-carbon metabolism, the return to a normal diet after weaning remained associated with growth and weight deficits [10,13]. Furthermore, we previously showed that the perinatal deficiency in methyl donors led to an increase of gastric ghrelin mRNA expression but also to a defect of the protein secretion, inducing a reduction of plasma ghrelin concentration in the deficient pups [13]. The central systems regulating energy homeostasis contain a regionalized and interconnected neural network, and most of the neuronal projections and synaptic connections between the hypothalamic nuclei develop within the first weeks after birth [28]. During this period, the integration of peripheral metabolic signals (e.g., ghrelin, leptin, insulin, peptide YY), especially to the arcuate nucleus, is essential for the harmonious and functional organization of the hypothalamus [29]. Moreover, the development of axonal projections from the arcuate nucleus to the paraventricular nucleus of the hypothalamus is highly sensitive to changes in the nutritional environment [18,30,31]. In the rat, plasma leptin is detectable at embryonic day 19 and plasma ghrelin at embryonic day 17 [32,33,34]. Thus, if during the perinatal period the integration of these signals to the arcuate neurons is disrupted, it will disturb the production of the neuropeptides, respectively POMC (which will produce by cleavage the anorexigenic α-Msh peptide), and the orexigenic neuropeptides NPY and AgRP [35]. This signaling defect will permanently affect the neuronal projections from the arcuate nucleus to the various functional hypothalamic areas possessing receptors for these neuropeptides (Y1R, MC4R, etc.), as observed in leptin-deficient mice (Ob/Ob) [36,37]. The capacity of nutritional status to alter the developing hypothalamus has been demonstrated in rodent models of maternal obesity or of protein starvation [23]. For example, maternal high-fat feeding increases hypothalamic cell proliferation in rat embryos, resulting in higher numbers of neurons containing orexigenic neuropeptides in the paraventricular nucleus and the lateral hypothalamic area [30], whereas gestational low-protein diet produces hypoleptinemia, increased hypothalamic gene expression of the orexigenic neuropeptides NPY and AgRP, and decreased expression of the anorexigenic neuropeptides POMC and CART [35]. These results suggest that the early nutritional environment can affect the development of energy balance circuits, and these effects appear to be mediated, to some extent, by abnormal leptin, ghrelin, and insulin secretion and/or signaling during critical periods of fetal and/or postnatal development.

The present study showed that methyl donor deficiency leads to an increase of ghrelin mRNA and protein expression in the hypothalamus, as previously shown in the stomach [13]. However, since the gastric gland is the major site of ghrelin production, plasma ghrelin is dramatically reduced due to altered secretion of the protein. We previously showed that methyl donor deficiency is accompanied by a 50% reduction of global DNA methylation in 20-day-old rat embryos [25]. The observed increased mRNA expression of ghrelin can be due to the demethylation condition but, to our knowledge, there is no available data demonstrating that the promoter of the ghrelin gene is regulated by methylation. We report for the first time that perinatal methyl donor deficiency also affects other peripheral metabolic signals, leading to increased plasma concentration of the anorexigenic peptide YY and decreased plasma concentration of leptin. In our model, plasma insulin was also reduced but presented a large variability between pups (data not shown).

The induction of vitamin B9 deficiency in rat and mouse embryonic cell lines, respectively corresponding to hypothalamic ghrelin cells and NPY cells, allowed us to depict the specific effect of folate deficit on gene expression without interference or regulation by other neurons or peripheral signals. The observed folate-related effects would mainly reflect epigenetic regulations such as promoter or histone methylation or post-transcriptional regulation by microRNA, as previously documented by our laboratory [11,25,38]. In these cell models, we observed specific effects of folate deficiency on mRNA expression depending on the neuropeptide secreted by the cells. Specifically, folate deficiency led to decreased NPY expression and to overexpression of AgRP mRNA, along with reduced expression of the leptin and insulin receptors, whereas ghrelin overexpression was associated with reduced expression of these receptors.

In vivo data in 21-day-old deficient pups differed from those obtained in vitro due to the presence of cell–cell regulations and peripheral metabolic signaling. Surprisingly in the hypothalamus of deficient pups, both orexigenic (ghrelin/NPY mRNA) and anorexigenic pathways (POMC mRNA) appeared to be stimulated. However, immunohistochemical analyses of the arcuate nucleus revealed that NPY mRNA in excess was not translated, since the protein labelling was lower than control. This could be the result of specific microRNA regulation as documented in the literature [39,40]. Recently, Yang and colleagues showed that an excess intake (10-fold) of folic acid during gestation is linked to increased food intake and obesity in male rat offspring post-weaning [41]. In their model, high gestational folic acid levels increased the number of mature NPY-positive neurons in the hypothalamus of the male offspring, which can contribute to increased food intake and higher body weight later in life. Accordingly, we observed the contrary in our model of gestational B9/B12 restriction, since the results showed a predominance of the anorexigenic pathway, with increased plasma peptide YY, increased hypothalamic POMC, and, as previously shown [10,13], definitive growth and weight deficits in spite of the return to a normal diet at weaning.

The hypothalamus develops early during gestation and continues during the postnatal period [42]. These developmental windows represent important periods of vulnerability during which environmental alterations may lead to abnormal hypothalamic development. We recently reported that an adapted maternal folate supplementation from the beginning of the third week of gestation till weaning alleviates methyl donor deficiency-associated birth defects in the rat progeny [25,27]. This perinatal period corresponds to a peak of brain maturation with the occurrence of numerous neuronal adaptative changes, like synaptogenesis, circuit refinement and plasticity. In the present study, we observed that maternal administration of folic acid tended to rectify peripheral metabolic signaling and central neuropeptide and receptor expression, leading to reduced growth retardation, weight deficit and brain atrophy. This suggests that perinatal folate supplementation may beneficially influence the developmental plasticity and epigenetic programming of the hypothalamic circuits.

## 4. Materials and Methods

### 4.1. Cell Cultures

mHypoE46 (mouse) et rHypoE11 (rat) neuronal cell lines purchased from American Type Culture Collection (ATCC) were conditionally immortalized by transfer of a temperature-sensitive simian virus 40 large tumor (SV40 T) antigen to primary hypothalamic neuronal cell cultures obtained from fetal mice on embryonic days E15, E17 and E18, and from E18 fetal rats [43]. Cells were cultivated in 1× Dulbecco’s modified Eagle’s medium (DMEM, D5796, Sigma-Aldrich, Saint-Quentin Fallavier, France) with 10% fetal bovine serum (CVFSVF0001, lot: S52751-2262, Eurobio, Courtaboeuf, France), 1% penicillin/streptomycin (P4333, Sigma-Aldrich) and maintained at 37 °C with 5% CO_2_. For homogeneity, the same lot of serum was used throughout all experiments. The cells grew to form a monolayer culture, attached to the culture plate and were split when they reached 70–90% confluence, with a plate ratio of 1:5. Because standard DMEM does not contain vitamin B12, methyl donor deficiency was induced by using a poor medium (DMEM D2429, Sigma-Aldrich) lacking B9 (folic acid), with addition of 2 mM glutamine (G7513, Sigma-Aldrich), 3.7% sodium bicarbonate (S8761, Sigma-Aldrich), 0.35% glucose (G8769, Sigma-Aldrich), 10% fetal bovine serum and 1% penicillin/streptomycin. Cells were kept in B9-free conditions for 24 or 48 h before subsequent analyses.

### 4.2. Animals and Tissue Collection

Animal experiments were conducted by using a validated rat model of early methyl donor deficiency, as previously described [10,44]. They were performed according to the European guidelines (2010-63-UE), and were approved by the local University Research Ethics Board (CELMEA, approval APAFIS#5509-2016053112249550, March 31, 2017). Wistar rats (Charles River, l’Arbresle, France) were maintained in an accredited facility according on a 12-h light/dark cycle with access to food and water ad libitum. One month prior to mating, females (*n* = 5 per experimental group matched to one male for two females for mating) were fed either a standard diet (Maintenance diet M20, Scientific Animal Food and Engineering, Villemoisson-sur-Orge, France) or a diet deficient in folate and vitamin B12 and lowered in choline (Special Diet Service, Saint-Gratien, France). Methionine content (~0.4%) was similar in both diets. For maternal supplementation, folic acid (the synthetic form of folate, Sigma-Aldrich) diluted in condensed milk was given per os. at the dose of 3 mg/kg per day in a final volume of 1 mL to dams from embryonic day 13 to postnatal day 21 (weaning). Matched control dams received the same volume of vehicle (i.e., 1 mL condensed milk) over the same period, as previously described [25] (refer to Figure S1 for the experimental design). Whatever the maternal diet, all pups of both sexes (15 ≤ *n* ≤ 40 issued from at least three different litters per experimental group) were euthanized between 8:00 and 10:00 by excess isoflurane at 21 days of age and blood was withdrawn for subsequent plasma measurements. Individuals were weighed and evaluated morphologically. The brains were rapidly collected, weighed, and the hypothalamus was carefully dissected before freezing in liquid nitrogen and storage at –80 °C. For immunochemistry, brains were fixed with 4%-paraformaldehyde 4% (24–48 h) at 4 °C, dehydrated and included in paraffin. Sagittal brain sections (12 µm) were generated at the level of hypothalamus by means of a microtome. They were then mounted onto glass slides and stored at ambient temperature.

### 4.3. Plasma Assays

Plasma concentrations of vitamins B9 and B12 were determined using a radioisotope dilution assay (simulTRACSNB; ICN Pharmaceuticals, Versailles, France) as previously reported [45]. Concentrations of homocysteine were measured by high-performance liquid chromatography (Waters, St. Quentin, France) coupled with mass spectrometry (Api 4000 Qtrap; Applied Biosystems, Courtaboeuf, France) [46].

Plasma levels of total ghrelin was measured in duplicate after appropriate dilution by specific radioimmunoassays using commercial kits (RK-031-31; Phoenix Europe GmbH, Karlsruhe, Germany,), as previously described [13]. Leptin, peptide YY and insulin were measured using the MILLIPLEX Rat Metabolic Hormone Panel (RMHMAG-84K, Millipore, Fontenay-sous-Bois, France).

### 4.4. RNA Extraction and Quantitative RT-PCR

RNA was purified from cell cultures by using a commercial kit (NucleoSpin^®^ RNA Plus, Macherey Nagel, Düren, Germany). In the case of rat hypothalamus tissues, RNA extraction was performed with TRIzol^®^ (Invitrogen, Cergy-Pontoise, France) according to the manufacturer’s instructions. The concentration and purity of RNA were determined at 260/280 nm by using a nanodrop spectrophotometer Multiskan GO (Thermo Scientific, Fisher, Illkirch, France).

RNA (300 ng) was then subjected to a two-step RT-qPCR using the PrimeScript™ RT Master Mix and SYBR^®^ Premix Ex Taq^®^ (Takara, Ozyme, Saint-Cyr-l’Ecole, France) following the manufacturer’s instructions. Primers are detailed in Table 2 and were purchased from Eurogentec (Liège, Belgium). The products of amplification were analyzed by agarose gel electrophoresis to confirm amplicon size and primer specificity (a single band at the expected size). Cycle threshold (*C*t) was determined from each sample and real-time PCR amplification efficiencies were expressed by calculating the ratio of crossing points of amplification curves. The expression of genes of interest was normalized to those of GAPDH/RPS29 for the rat species and Pol2/RPS29 for the mouse species using the 2^−ΔΔ*C*t^ method.

### 4.5. Immunohistochemistry

Immunohistological analyses were performed on brain sections at the level of the arcuate nucleus of the hypothalamus according to the Paxinos and Watson rat brain atlas [47]. Nonspecific binding sites were blocked in phosphate-buffered saline containing 1% bovine serum albumin (BSA) and incubation was performed overnight with an antibody against one of the following proteins: NPY (rabbit polyclonal, 1/200, Cell Signaling Technology, Ozyme, Saint-Cyr-l’Ecole, France), ghrelin (rabbit polyclonal, 1/200, Millipore, Fontenay-sous-Bois, France). Cell nuclei were counterstained with the DNA fluorochrome 4,6-diamidino-2-phenylindole (DAPI, Sigma-Aldrich). After a washing step, immunoreactivity was assessed by incubation in the presence of an appropriate secondary anti-IgG antibody conjugated to AlexaFluor for 1 h at 25 °C (1/1000, Life Technologies, Saint-Aubin, France). Control experiments were conducted by omitting the primary antibody. Immunofluorescence visualization and image acquisition (×20 and ×60 magnification) were performed with a confocal microscope (Nikon Instruments, Champigny sur Marne, France) and analyzed by Cell^®^ software (version 3.1, Olympus, Rungis, France).

### 4.6. Statistical Analysis

Data were analyzed with StatView 5 software for Windows (version 5.0, SAS Institute, Berkley, CA, USA). They were compared by using one-way analysis of variance (ANOVA) with Fisher’s test. A *p*-value < 0.05 was considered to indicate significance.

## 5. Conclusions

The results of the present study suggest that early methyl donor deficiency can impair the hypothalamus development, with altered expression of neuropeptides and related receptors regulating eating behavior and energy homeostasis, leading to growth retardation. If maternal supplementation with folic acid during the perinatal period could not fully reverse the deleterious consequences of deficiency, it was shown to exert beneficial effects.

## Figures and Tables

**Table 1:** Effects of the maternal dietary regimen on plasma concentrations of folate, vitamin B12, homocysteine, body weight and brain weight in 21-day-old rat pups.

	Plasma Folate (nmol/L)	Plasma Vitamin B12 (pmol/L)	Plasma Homocysteine (µmol/L)	Body Weight (g)	Brain Weight (g)
**21-d Control-vehicle**	53.1 ± 7.5	876.6 ± 94.1	6.7 ± 1.8	48.6 ± 3.2	1.43 ± 0.20
**21-d MDD-vehicle**	23.8 ± 7.6 **	249.4 ± 211.6 **	23.3 ± 7.1 **	22.1 ± 2.0 **	1.01 ± 0.29 **
**21-d Control + B9**	103.2 ± 42.7 **	899.3 ± 134.6	4.2 ± 2.4	48.4 ± 3.7	1.51 ± 0.11
**21-d MDD + B9**	94.1 ± 46. 4 **/°°	249.7 ± 102.2 **	4.5 ± 3.7 **/°°	27.2 ± 3.1 **/°°	1.32 ± 0.36 **/°°

Data are means ± SD and were obtained from 15 ≤ *n* ≤ 40 individuals. Statistically significant differences: ** *p* < 0.01, with respective control; °° *p* < 0.01, between MDD and MDD + B9 (MDD = methyl donor deficiency).

**Table 2:** Sequences of primers used for quantitative PCR.

Gene	Forward	Reverse	Species
*Neuropeptide Y*	AGATCCAGCCCTGAGACACT	TTCAAGCCTTGTTCTGGGGG	Rat
*Neuropeptide Y receptor 1*	TGC-TAC-TTC-AAG-ATA-TAC-GTT-CGC	ACG-ATG-GAG-AGC-AGC-ATG-AC	Rat
*Insulin receptor*	GGA-CCA-GGC-ATC-CTG-TGA-AA	ATC-CTG-CCC-GTC-AAA-CTC-TG	Rat
*Leptin receptor*	CCC-CCA-CTG-AAA-GAC-AGC-TT	GGC-TTC-ACA-ACA-AGC-ATG-GG	Rat
*Pro-opiomelanocortin*	CGA-CGG-AGG-AGA-AAA-GAG-GTT	CTG-AGG-CTC-TGT-CGC-GGA-A	Rat
*Insulin growth factor receptor 1*	AAG-GCC-AGA-GGT-GGA-GAA-TAA	TAC-CAT-GCA-GTT-CCG-AGC-AG	Rat
*Ghrelin*	CCA-AGA-AGC-CAC-CAG-CTA-AA	CTG-ATT-TCC-AGC-TCC-TCC-TC	Rat
*Neuropeptide Y receptor 1*	Reference: qMmuCIP0029884 (BioRad, Marnes-la-Coquette, France)		Mouse
*Leptin receptor*	Reference: qMmuCID0015266 (BioRad)		Mouse
*Agouti-related protein*	CGG-AGG-TGC-TAG-ATC-CAC-AGA	AGG-ACT-CGT-GCA-GCC-TTA-CAC	Mouse
*Insulin growth factor receptor 1*	GCA-CCA-ATG-CTT-CAG-TCC-CT	TTG-GAG-CAG-TAG-TTG-TGC-CG	Mouse
*Insulin receptor*	AGA-TGT-CCC-ATC-AAA-TAT-TGC-CA	CAT-CCG-GCT-GCC-TCT-TTC-TC	Mouse

## Supplementary Materials

The following are available online at https://www.mdpi.com/1422-0067/20/20/5097/s1, Figure S1.
