# Supplementary material for: Methyl Donor Deficiency during Gestation and Lactation in the Rat Affects the Expression of Neuropeptides and Related Receptors in the Hypothalamus
Source: Int J Mol Sci. 2019 Oct 14;20(20):5097. doi: 10.3390/ijms20205097 (PMC6829491; doi:10.3390/ijms20205097)
Supplement: Supplementary file 1 [file ijms-20-05097-s001.pdf]

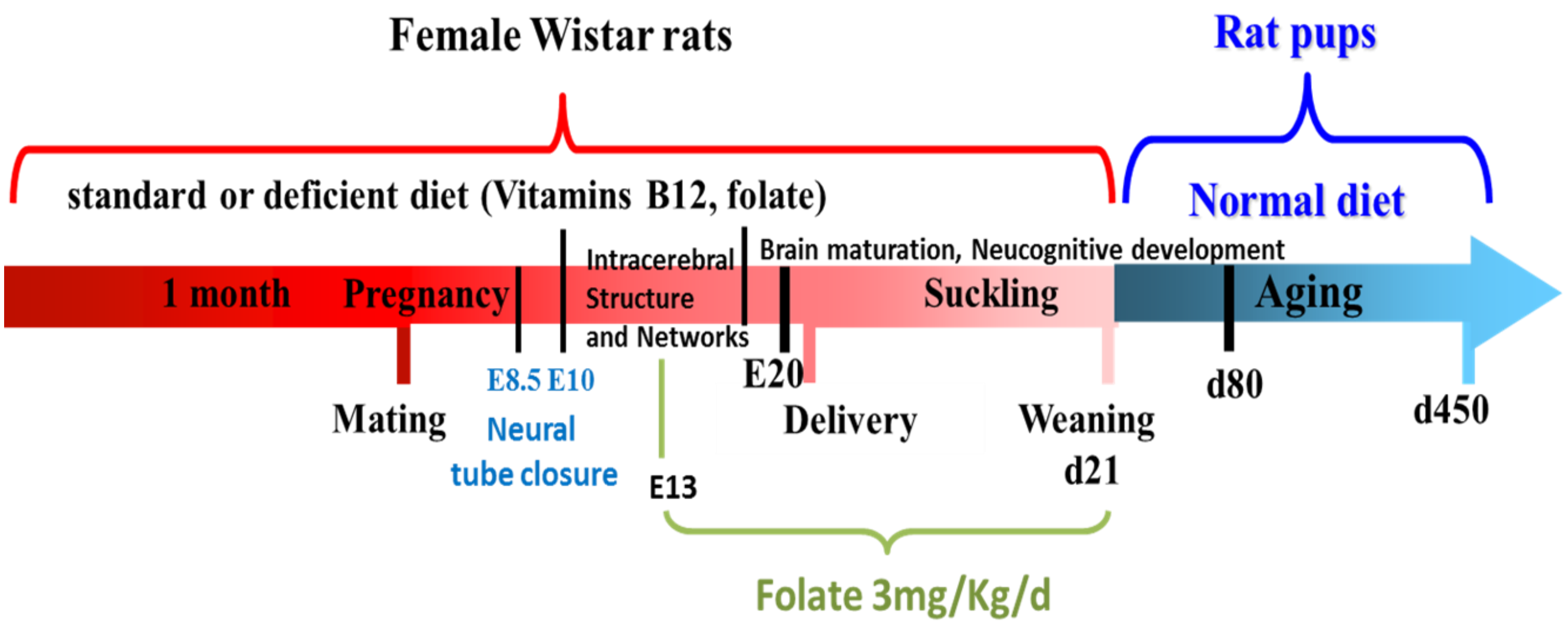

Summary diagram illustrating the experimental design of the animal study.

One month before mating, female rats received either standard diet or a diet deficient in vitamin B12 and folate. The corresponding regimen was assigned until weaning of the offspring. Between embryonic day 13 (E13) and postnatal day 21 (d21 corresponding to weaning) some dams of both experimental groups received either folic acid or condensed milk (vehicle).
